# Transcriptome Analysis of Leaf Tissue of *Raphanus sativus* by RNA Sequencing

**DOI:** 10.1371/journal.pone.0080350

**Published:** 2013-11-12

**Authors:** Libin Zhang, Haibo Jia, Yongtai Yin, Gang Wu, Heng Xia, Xiaodong Wang, Chunhua Fu, Maoteng Li, Jiangsheng Wu

**Affiliations:** 1 School of Life Science and Techonology, Huazhong University of Science and Technology, Wuhan, Hubei, China; 2 Institute of Biology and Medicine, Wuhan University of Science and Technology, Wuhan, Hubei, China; 3 National Key Laboratory of Crop Genetic Improvement, Huazhong Agricultural University, Wuhan, China; University of Georgia, United States of America

## Abstract

*Raphanus sativus* is not only a popular edible vegetable but also an important source of medicinal compounds. However, the paucity of knowledge about the transcriptome of *R. sativus* greatly impedes better understanding of the functional genomics and medicinal potential of *R. sativus*. In this study, the transcriptome sequencing of leaf tissues in *R. sativus* was performed for the first time. Approximately 22 million clean reads were generated and used for transcriptome assembly. The generated unigenes were subsequently annotated against gene ontology (GO) database. KEGG analysis further revealed two important pathways in the bolting stage of *R.sativus* including spliceosome assembly and alkaloid synthesis. In addition, a total of 6,295 simple sequence repeats (SSRs) with various motifs were identified in the unigene library of *R. sativus*. Finally, four unigenes of *R. sativus* were selected for alignment with their homologs from other plants, and phylogenetic trees for each of the genes were constructed. Taken together, this study will provide a platform to facilitate gene discovery and advance functional genomic research of *R. sativus.*

## Introduction

The *Brassicaceae* include many vegetables with great economic value, one of the most familiar of which is the root vegetable radish (*Raphanus sativus*). Widely cultivated in the Far East, including China, Korea and Japan, *R. sativus* contains many varieties with different sizes, colors, and planting times depending on their intended use. Besides being an important edible crop, *R. sativus* is also an important source of medicinal compounds [1]. For example, the roots of *R. sativus* contain multiple peroxidases that could be used for a range of medicinal applications [2]. Recently, high-throughput sequencing was used to reveal miRNA and transcriptome profiles of *R. sativus* root, which will expand the understanding of *R. sativus* miRNA function in multiple biological processes [3,4]. Similar to *Brassica rapa*, the genome of *R. sativus* is relatively small and diploid (2n = 18). However, given that only one tissue (root) transcriptome profiling of *R. sativus* has been reported, the very limited availability of useful genomic information for *R. sativus* has greatly impeded progress in improving our understanding of the functional genomics and medicinal potential of *R. sativus.*

mRNA-Seq technology has emerged as an effective transcriptome analysis tool that is now widely used in biological research, clinical field, and drug development. mRNA-Seq provides a more sensitive and accurate way of analyzing transcriptome profiles than microarray and other technologies [5-7]. For instance, mRNA-Seq can systematically analyze transcriptional activity at the single-nucleotide level [8]. Accordingly, mRNA-Seq is widely used not only to detect transcriptome profiles but also to identify differentially expressed genes, discover unknown transcripts, and accurately identify cSNP (Coding Single Nucleotide Polymorphism) in multiple non-model plants [9-18]. More importantly, mRNA-seq does not rely on the availability of any genomic information for the species being studied. Therefore, mRNA-Seq has been widely used for transcriptome analysis of non-model organisms without genome information and to quantify the expression of genes of interest [19-26]. The *de novo* assembly of transcriptome data facilitates the functional genomic analysis of species for which a genome sequence is not available, and can also be used for genome annotation and the identification of microsatellite or SNP markers [27-32]. 

 In this study, we first applied mRNA-Seq technology to characterize the transcriptome profiles of leaf tissues in *R. sativus*. The Trinity program [19] was used for the *de novo* assembly of the *R. sativus* transcriptome, and a total of 28,410 unigenes were generated. All of the unigenes were annotated and analyzed by using the BLAST algorithm (http://www.ncbi.nlm.nih.gov/blast/Blast.cgi) to identify the unigene sequences through comparisons with various protein and nucleotide databases. Importantly, KEGG analysis showed that spliceosome and alkaloid biosynthesis were the important pathways in the bolting stage of *R. sativus* leaf tissues. Furthermore, approximately 6,000 SSRs with various motifs were developed in the assembled unigene library. Finally, we aligned four *R. sativus* unigenes with their homologs from other species within the *Brassicaceae*, and used the homology data to build phylogenetic trees using the neighbor-joining method.

Taken together, the *de novo* assembly and further annotation and analysis of transcriptome will provide comprehensive genome resources that will promote further functional genomic analysis of *R. sativus* and its closest relatives. 

## Materials and Methods

### Plant material and RNA isolation

The *R. sativus* big root radish was provided by Prof. Jiangsheng Wu (Huazhong Agricultural university, China), which was cultivated in the experiment field of Huazhong University of Science and Technology in 2012. The leaves were harvested after it had grown for three months and stored at -80°C. Total RNA was isolated by using TRIzol (Invitrogen) according to the manufacturer’s instructions, and DNase I (Promega) was used to remove contaminating genomic DNA. Total RNA was quantified by using a NanoDrop spectrophotometer (Thermo Fisher Scientific, Inc.), and the purity of the total RNA was detected by measuring both the A260/280 and A260/230 ratios. Furthermore, Agilent 2100 Bioanalyzer (Agilent Technologies, Inc.) was utilized to assess the integrity of the RNA samples.The purified RNA was dissolved in RNase-free water and stored in a -80°C freezer until subsequent analysis. 

### cDNA preparation and sequencing

The TruSeqTM RNA Sample Preparation Kit (Illumina, Inc.) was used to construct a cDNA library for sequencing of the *R. sativus* transcriptome. In brief, poly-A mRNA was purified from 10 μg of total RNA using oligo (dT) magnetic beads (NEB). The purified poly-A mRNA was then fragmented into fragments 200-500 bp in sizeThe fragmented mRNA pieces were used for the synthesis of first-strand cDNA with hexamer primers and reverse transcriptase (Promega), and then DNA polymerase I and RNase H were used to synthesize the second-strand cDNA. The cDNA fragments were then purified, end-repaired, A-tailed, and ligated to index adapters (Illumina). The ligated products were subsequently PCR-amplified to generate the final cDNA libraries. The cDNA library was sequenced using the Illumina GA IIX sequencing platform and the average length of sequenced reads was 75 nt.

### De novo transcriptome assembly

After sequencing of the cDNA library, base-calling using Illumina Pipeline Software was used to transform the raw image data generated into sequence information. The clean reads were obtained and deposited in NCBI Sequence Read Archive (SRA) Sequence Database with accession number SRP022926 by trimming adaptor sequences and removing empty reads and ambiguous nucleotides (‘N’ in the end of the reads). The Trinity program [19] was further used to assemble the clean reads to generate non-redundant unigenes. Briefly, reads with overlaps were assembled to generate contigs, which were joined into scaffolds that were further assembled through gap filling to generate sequences called unigenes. There is no single absolutely optimal k-mer length for transcriptome assembly because transcriptome coverage is highly variable owing to differential gene expression in cells. Here, a default k-mer size of 25 was set for the *de novo* assembly of the *R. sativus* transcriptome. Default values were used for all other parameters, and the assembled unigenes used for further annotation were all at least 200 bp in length.

### Transcriptome annotation

All of the assembled unigenes were analyzed using the BLAST algorithm to search for homologs in the NCBI non-redundant protein (Nr), Swissprot, Pfam and Trembl databases. The BLASTX algorithm was used to identify homologous sequences with an E-value cut-off of 10^-5^. Based on the BLAST hits identified by interrogation of the Nr and Swiss-Prot databases, GO (Gene Ontology) annotation was performed using BLAST2GO [33] to obtain cellular component, molecular function, and biological process terms. The Kyoto Encyclopedia of Genes and Genomes (KEGG) database is used extensively to reveal molecular interaction network and metabolic pathways [34]. KEGG pathways annotation was performed by mapping the sequences obtained from BLAST2GO to the contents of the KEGG metabolic pathway database (http://david.abcc.ncifcrf.gov/).

### RT-PCR validation of unigenes

Total RNA from *R. sativus* leaves was reverse-transcribed by using SuperScript III Reverse Transcriptase (Invitrogen) and oligo(dT)_18_. Five assembled unigenes were randomly selected for RT-PCR validation. Forward (Fwd) and reverse (Rev) primers were designed using Primer3. The sequences of primers used for validation of assembled *R. sativus* unigenes were also listed in Table S1.

### SSR prediction

Simple sequence repeats (SSRs) provide the most popular molecular-marker method used in quantitative trait loci (QTL) exploration, genetic analysis, and high-throughput genome mapping [35-37]. Mining of the SSRs present in the assembled unigenes of *R. sativus* was performed as described previously [32]. Briefly, five categories of SSRs, including those with dinucleotide, trinucleotide, tetranucleotide, pentanucleotide, and hexanucleotide motifs, were classified. For each category of SSR, the minimum number of contiguous repeat units is five. Statistical analysis was used to investigate the number of SSRs with each type of motif and the distribution of the lengths of repeat units. 

### Phylogenetic analysis of unigenes

The *R. sativus* unigenes identified after transcriptome assembly were first aligned with the same genes of *Brassicaceae* plants retrieved from Genbank after interrogation of the database using CLUSTAL X version 2.0 [38]. Subsequently, based on the nucleotide sequence alignments, MEGA4.0 software was used to generate phylogenetic trees of *R. sativus* unigenes and their homologs from other plants, using the neighbor-joining approach [39]. The phylogenetic trees were furnished with 1,000 pseudoreplicate bootstrap values at each node. The accession numbers for the sequences of U2AF35, GIGANTEA, EMB2369 and ATIMD2 unigenes that were included in the tree were listed as follows: *R. sativus*: comp20129_c0_seq1; *R. sativus*: comp7714_c0_seq1; *R. sativus*: comp11084_c0_seq1; and *R. sativus*: comp28243_c0_seq1(Table S2).

## Results and Discussion

### mRNA-Seq and de novo transcriptome assembly

**Figure 1:**
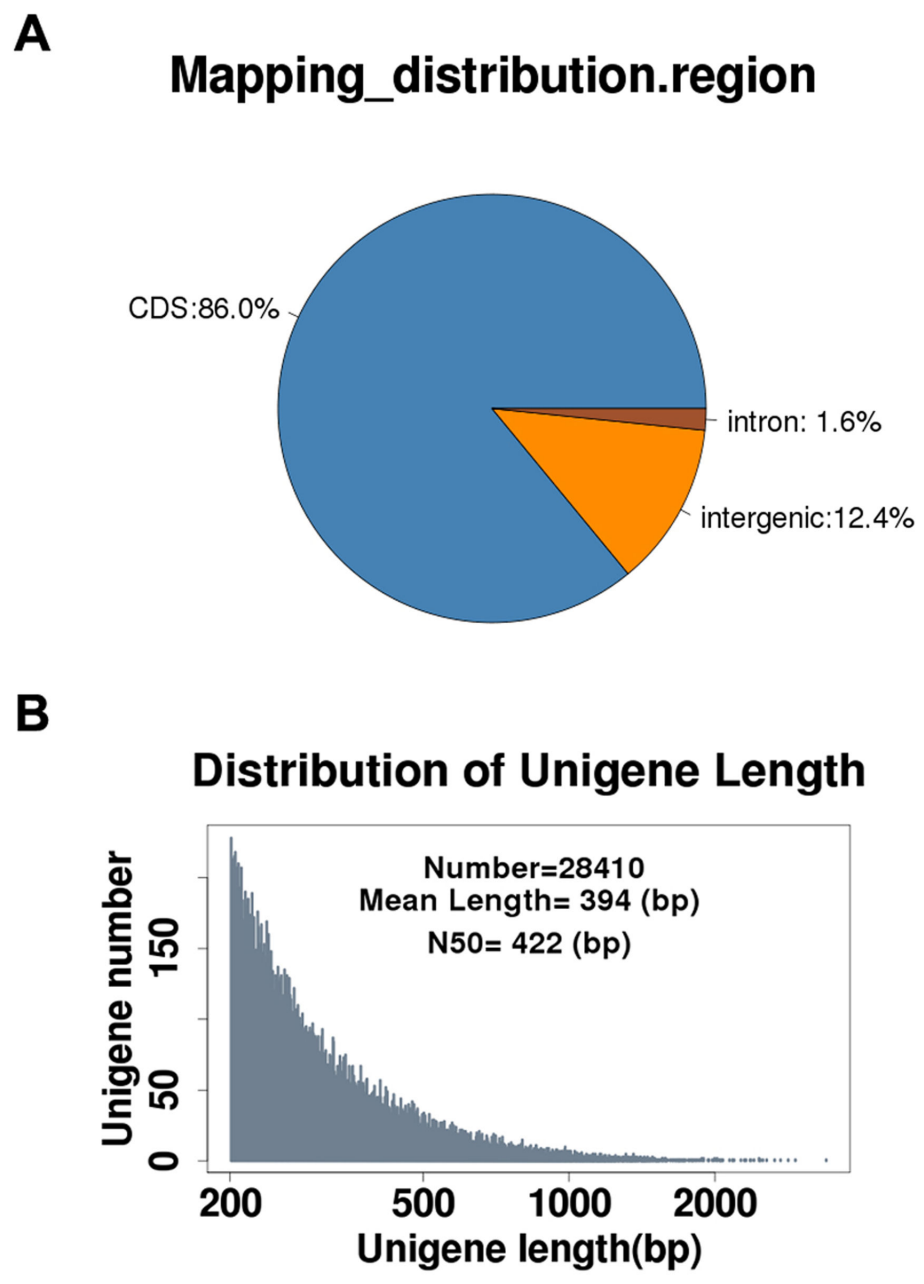
mRNA-seq of *R. sativus* leaves and *de* *novo* assembly of *R. sativus* transcriptome. (A).The distribution of clean reads on *Brassica rapa* genome. (B).The *de* *novo* assembly result of *R. sativus* transcriptome by Trinity program.

High-throughput sequencing technology has been used extensively to explore the transcriptome profiles of many non-model plant species. Here, we report the use of Solexa high-throughput sequencing technology to determine transcriptome profiles of *R. sativus*. A cDNA library of mRNAs from *R. sativus* was sequenced using the Illumina GA II X instrument, and a total of 27,976,916 raw reads were produced. A total of 21,978,870 clean reads were generated after removing adaptor sequences and low-quality reads. All clean reads were mapped to the *B. rapa* genome using Bowtie program. The result revealed that the clean reads were mainly distributed in the CDS (coding sequence) region (Figure 1A), which demonstrated that the mRNA-Seq results were highly reliable. The Trinity program was then used for *de novo* assembly of all clean reads, with generation of a total of 28,410 unigenes with an average length of 394 bp and an N_50_ of 422 bp (Figure 1B and Table 1). Among the unigenes, the shortest and longest unigenes are 200 bp and 3,392 bp, respectively. Moreover, 19,107 unigenes were within the 200–400 bp range, and 8,499 unigenes were within the 400–1,000 bp range. The observation that only 804 of the unigenes were longer than 1,000 bp might be explained by the documented roles of ubiquitous alternative splicing events and repeats element in perturbing *de novo* assembly of longer transcripts [40]. To evaluate the accuracy of *de novo* assembly of *R. sativus*, the clean reads obtained using mRNA-Seq were mapped to the unigenes. The results showed that 68.2% (14,994,188 clean reads) of clean reads could map to the assembled unigenes. Among 14, 994,188 mapped reads, 11,818,198 (78.8%) reads uniquely matched to the unigenes and 3,175,990 (21.2%) mapped to multiple locations on unigenes. We randomly selected five assembled unigenes of *R.sativus* for RT-PCR validation. The RT-PCR data indicated that all five selected unigenes of *R.sativus* got right amplifications (Figure S1). In addition, given that both *B. rapa* and *R. sativus* belong to the *Brassicaceae* family, we assume that the *R. sativus* geneome contains similar number of genes as *B. rapa*, which has 41,174 protein coding genes with an average length of 2,015 bp [41]. In order to estimate the level of transcripts coverage of *R.sativus*, indirect evaluation was performed by comparing the number of unigenes (28,410) and the average length (394 bp) to the *B. rapa* genes. The assembly generated in this study was estimated able to cover 13.5% of the *R. sativus* transcriptome. 

**Table 1:** Assembly summary of leaf unigenes of *Raphanus sativus*.

**Assembly**	**Number**
Number of raw reads	27,976,916
Number of used reads	21,978,870
Total Unigenes generated	28,410
N_50_ length (bp)	422
Average Unigene length (bp)	394

Meanwhile, the sequencing data of root of *R.sativus* was downloaded from NCBI Sequence Read Archive and assembled using Trinity program. In order to investigate the tissue specificity of gene expression between root and leaf of *R.sativus*, we further performed a comparison of assembled unigenes between root and leaf. The result showed that a total of 50,700 unigenes of root of *R.sativus* were generated with an average length of 1,182 bp (Table S3). Furthermore, BLASTx analysis revealed that ^~^92% (26,225) leaf unigenes of *R.sativus* were perfectly matched to root unigenes of *R.sativus*, which indicated that the transcriptome profiles of root and leaf of *R.sativus* are very similar, whereas ^~^8% differential unigenes between root and leaf might be associated with tissue-specific gene expression.

Taking into account the results of this transcriptome assembly derived solely from one tissue of *R. sativus* (leaf) and that there is currently no available genome sequence information for *R. sativus*, our data suggest the suitability of mRNA-Seq for extensive and accurate assembly of plant transcriptomes.

### Annotation of the *R. sativus* transcriptome

**Figure 2:**
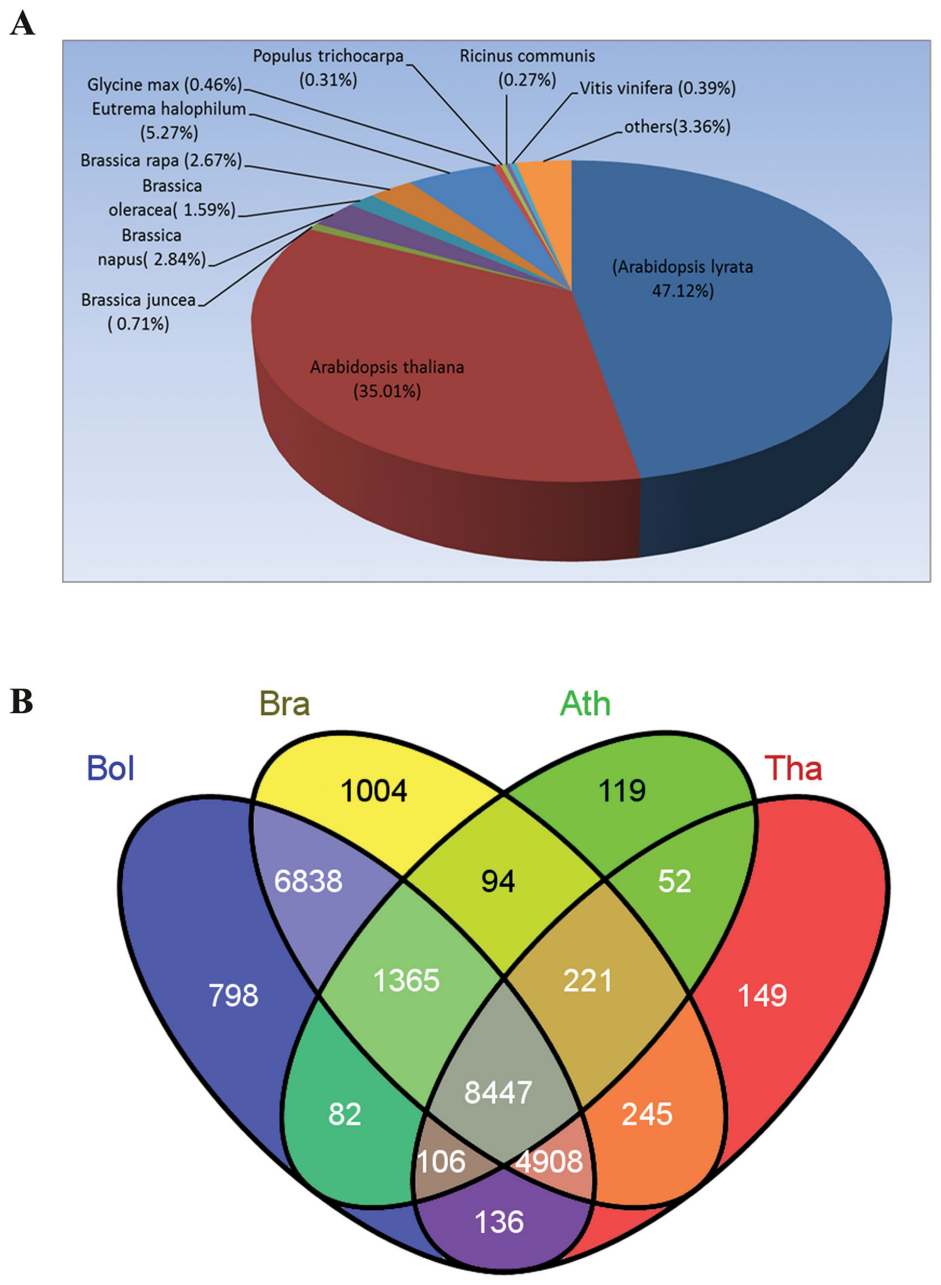
Comparative analysis of *R. sativus* unigenes with different species. (A). Homology analysis of *R. sativus* unigenes with multiple species. (B). Mapping result of *R. sativus* unigenes to CDS regions of familiar species. 28,410 *R. sativus* unigenes were mapped to the CDS sequences of *Arabidopsis thaliana*, *Brassica rapa*, *Brassica oleracea* and *Thellungiella halophila*. The obtained homologous unigene numbers were 10,486, 23,122, 22,680 and 14,264, respectively.

In order to assess and annotate the assembled unigenes, the 28,410 unigenes generated by Trinity were subjected to BLASTx similarity analysis (E-value cutoff of 10^-5^) involving interrogation of public protein databases, including the NCBI non-redundant protein (Nr) database, the Swiss-Prot protein database, Nt database, Pfam database and Trembl database. As a result, the mapping rate was between 54% and 91% (Table 2). Most of the unigenes (approximately 91%) were mapped to the Nt library. Importantly, mapping of 80% of the unigenes to the Nr library suggests that most of the unigenes can be translated into proteins. The mapping rates of unigenes against the Swissprot and Trembl protein databases were 54.8% and 80.3%, respectively. Distribution analysis based on BLASTx searches showed that the unigenes of *R. sativus* have homologs in numerous hit a lot of plant species (Figure 2A). Among the various plant species that have protein sequence information in GenBank, the unigenes of *R. sativus* had the highest number of hits to sequences from *Arabidopsis lyrata* (47.12%), followed by *Arabidopsis thaliana* (35.01%), *Eutrema halophilum* (5.27%), *Brassica napus* (2.84%), *Brassica rapa* (2.67%), and *Brassica juncea* (0.71%). In the meanwhile, in order to further verify the assembly results, we mapped unigenes to *Arabidopsis thaliana* (TAIR10_cds_20101028), *Brassica rapa* (v1.2), *Brassica oleracea* (v1.0) and *Thellungiella halophila* (*Thalophila*_173_cds.fa) CDS sequences using BLAT. Parameters of BLAT were set as default (minMatch=2, minScore=30, minIdentity=90, maxGap=2, tileSize=11 and stepSize=11). Among 28,410 unigenes, 10,486, 23,122, 22,680 and 14,26*4* were successfully mapped to CDS in *Arabidopsis thaliana*, *Brassica rapa*, *Brassica oleracea* and *Thellungiella halophila*, respectively (Figure 2B). The high similarity of *R. sativus* unigenes to genes from *A. lyrata and A. thaliana* suggests the possibility of using the genome of *A. lyrata* or *A. thaliana* as a reference for identifying differential gene expression patterns of mRNA-Seq data.

**Table 2:** Blast results of leaf unigenes of *Raphanus sativus*.

***Raphanus sativus* (Total unigenes: 28410)**	
**Database**	**Mapped Unigenes**
Pfam	18111(63.75%)
Nr	22885(80.55%)
Nt	25851(90.99%)
SwissProt	15576(54.83%)
Trembl	22812(80.30%)

### GO Classification of unigenes from *R. sativus*

**Figure 3:**
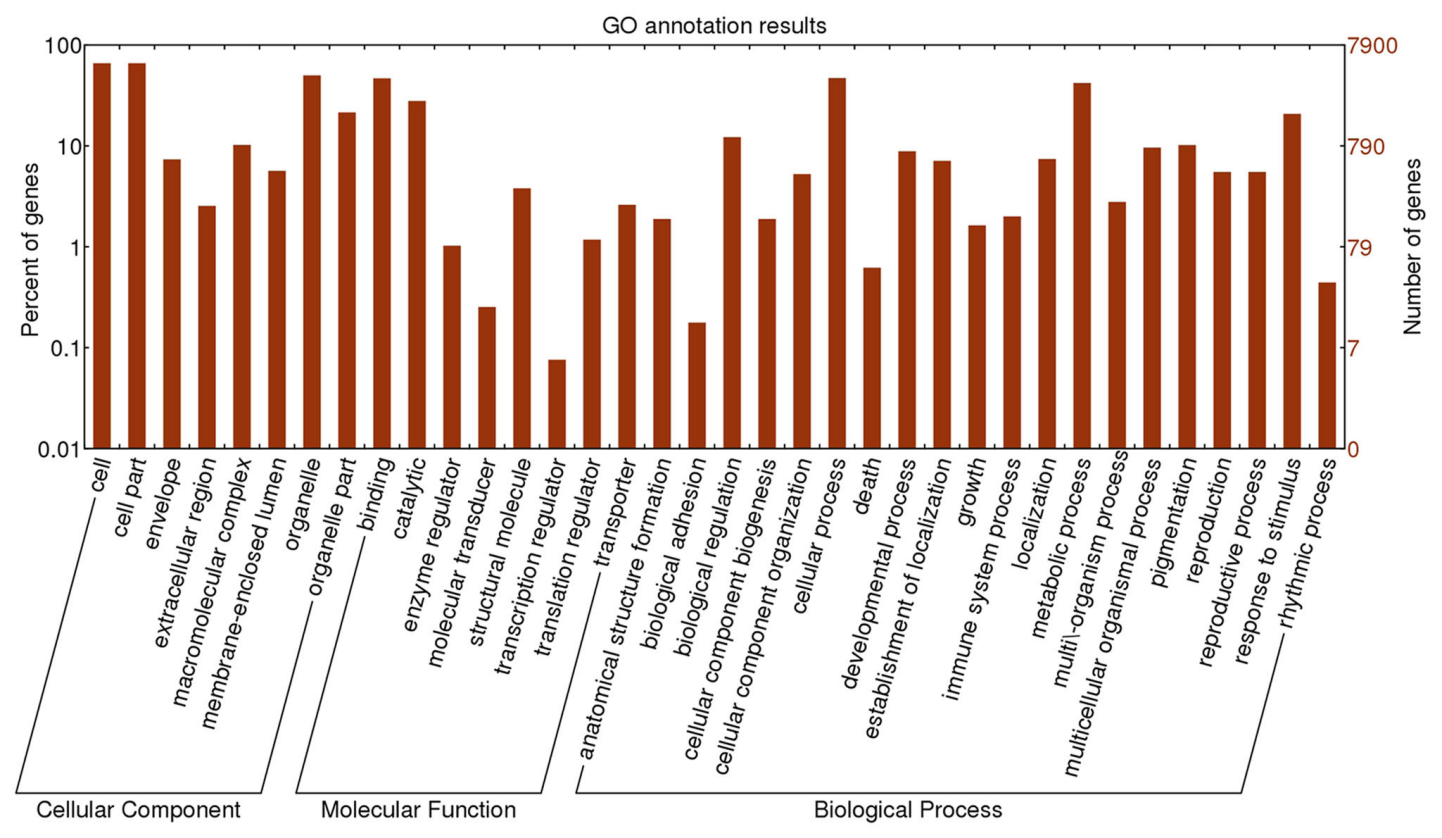
The annotation results of *R. sativus* transcriptome. GO (Gene Ontology) annotation results of assembled unigenes of *R. sativus*. The annotated unigenes were classified into Cellular Component, Molecular Function and Biological Process categories.

The *R. sativus* unigenes were subjected to BLASTx analysis against the Nr and Swiss Prot databases using a cutoff e-value of 10^-5^. This revealed that 23,032 unigenes (81.1%) had homologous sequences in the Nr and Swissprot databases. These unigenes were further annotated with GO terms using BLAST2GO. The annotated GO terms were classified into three categories: cellular component, biological process, and molecular function. The classification of annotated *R. sativus* unigenes is summarized in Figure 3, with 15,249 unigenes annotated with biological process, 6,601 with a molecular function, and 18,113 with a cellular component. In the cellular component category, unigenes were further classified into “cell” (5,216 terms), “cell part” (5,216 terms), “organelle” (3,947 terms), “organelle part” (1,698 terms) and other well-represented terms including “macromolecular complex” (807 terms), “envelope” (581 terms), “membrane-enclosed lumen” (447 terms), and “extracellular region” (201 terms). In the biological process category, the major GO terms were classified into “cellular process” (3,720 terms), “metabolic process” (3,321 terms), “response to stimulus” (1,641 terms), “biological regulation” (965 terms), “pigmentation” (805 terms), “multicellular organismal process” (760 terms), and “developmental process” (699 terms). In the molecular function category, the major GO terms were “binding” (3,693 terms) and “catalytic” (2,202 terms). However, the terms “structural molecule” (300 terms), “transporter” (206 terms), “translation regulator” (93 terms), “enzyme regulator” (81 terms), “molecular transducer” (20 terms), and “transcription regulator” (6 terms) were also represented.

### KEGG analysis of leaf unigenes of *R. sativus*

**Figure 4:**
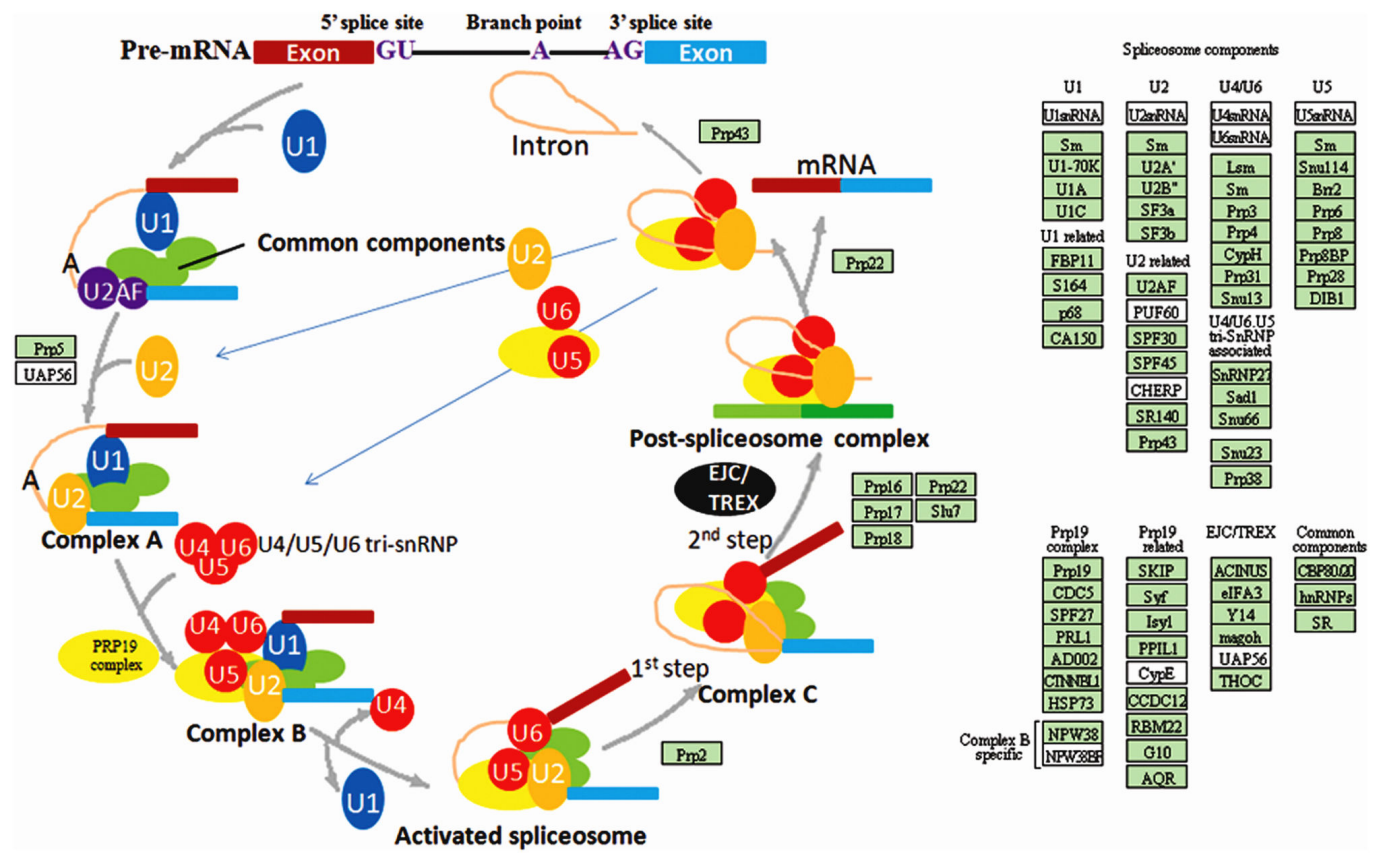
*R.sativus* unigenes (green rectangle) involved in spliceosome assembly pathway. Some *R.sativus* unigenes, such as Prp5, Prp2, Prp16, Prp12 and Prp43 etc, directly participated in the process of spliceosome assembly, while others made up the key components in spilceosome assembly including U1, U2, U4, U5 and U6 etc.

The KEGG database documents connections between known information on molecular interaction networks, such as information about metabolic pathways and the functions of gene products. To better understand the biological functions of *R. sativus* unigenes, the assembled *R. sativus* unigenes were further assigned to the biochemical pathways in the KEGG database (34). A total of 7158 unigenes were assigned to 117 KEGG biochemical pathways (Table S4). The top 3 pathways (ranked by p-value) included “spliceosome” (159 unigenes, p-value 5.12×10^-8^), “biosynthesis of alkaloids derived from histidine and purines” (254 unigenes, p-value 2.36×10^-4^), “Pentose phosphate pathway” (79 unigenes, p-value 7.5×10^-3^). As shown in Figure 4, multiple *R.sativus* unigenes (green rectangle) were involved in the process of spliceosome assembly. Some made up the key components of spliceosome assembly including U1, U2, U4, U5 and U6 etc. Some other unigenes, such as Prp5, Prp2, Prp16, Prp17, Prp18, Prp22, Slu7, Prp22 and Prp43, directly participated in the process of spliceosome assembly. As *R.sativus* in bolting stage grows very rapid, this result showed that versatile alternative splicing events may occur in the bolting stage of *R.sativus*, which suggested that alternative splicing regulation is an general approach to affect complex plant biological processes including plant development, disease resistance and stress responses etc. In the meanwhile, KEGG analysis showed that 254 unigenes of *R.sativus* were involved in the pathway of alkaloid biosynthesis (Figure S2). Given that alkaloids are crucial for defense against pathogens, this finding confirmed that *R. sativus* is an important source of medicinal compounds, which also suggested that alkaloid biosynthesis is a crucial physiological activity in the bolting stage of *R.sativus*. In the meanwhile, we performed the KEGG analysis of top 10 GO terms. Interestingly, the result showed that “alkaloid biosynthesis” and “Pentose phosphate pathway” were still major pathways and 95 unigenes were assigned to “Spliceosome” pathway (Table S5). Collectively, KEGG analysis of *R.sativus* unigenes shed light on the great potential of mRNA-Seq technology to identify novel genes in various plant metabolic pathways.

### SSR analysis of the *R. sativus* transcriptome

Simple Sequence Repeat (SSR) markers, also known as microsatellites, are repeating DNA sequences of 2–6 base pairs, which are widely used as molecular markers for genetic mapping and to analyze species diversity. Here, 6,295 SSRs were predicted in 2,755 unigenes, of which 2,048 unigenes contained multiple SSRs. The SSRs included 2,919 (46.4%) dinucleotide motifs, 3,137 (49.8%) trinucleotide motifs, 229 (3.6%) tetranucleotide motifs, 7 (0.1%) pentanucleotide motifs, and 3 (0.05%) hexanucleotide motifs (Table 3). Furthermore, 112 motifs were detected among all of the SSRs identified. The most abundant SSR motif was GA/AG/TC/CT (2,153 SSRs), followed by AAG/AGA/TTC/TCT/CTT/GAA/GAG (1,052 SSRs), and then AT/TA/TG/GT (572 SSRs). The distribution of the number of repeat units in all SSRs was also investigated (Table 3). The result revealed that most SSRs contained fewer than 10 repeat sequences, and no SSRs with more than 20 repeat sequences were observed. Although many SSR markers were identified in the *Brassicaceae* family, only a few SSR markers were reported in *R. sativus* [42-45]. Here, we predicted 6,295 SSRs from the assembled unigene library of *R. sativus*. These SSRs will likely be of value for genetic analyses of *R. sativus* and other related non-model plants. 

**Table 3:** SSRs distribution in the leaf unigenes of *Raphanus sativus*.

**Type**	**Repeats Number (<6)**	**Repeats Number (6-10)**	**Repeats Number (10-20)**	**Total**
Di-nucleotide	1461	1370	88	2919
Tri-nucleotide	1904	1230	3	3137
Tetra-nucleotide	214	15	0	229
Penta-nucleotide	4	3	0	7
Hexa-nucleotide	3	0	0	3

### Phylogenetic analysis of interesting genes in *R. sativus*

**Figure 5:**
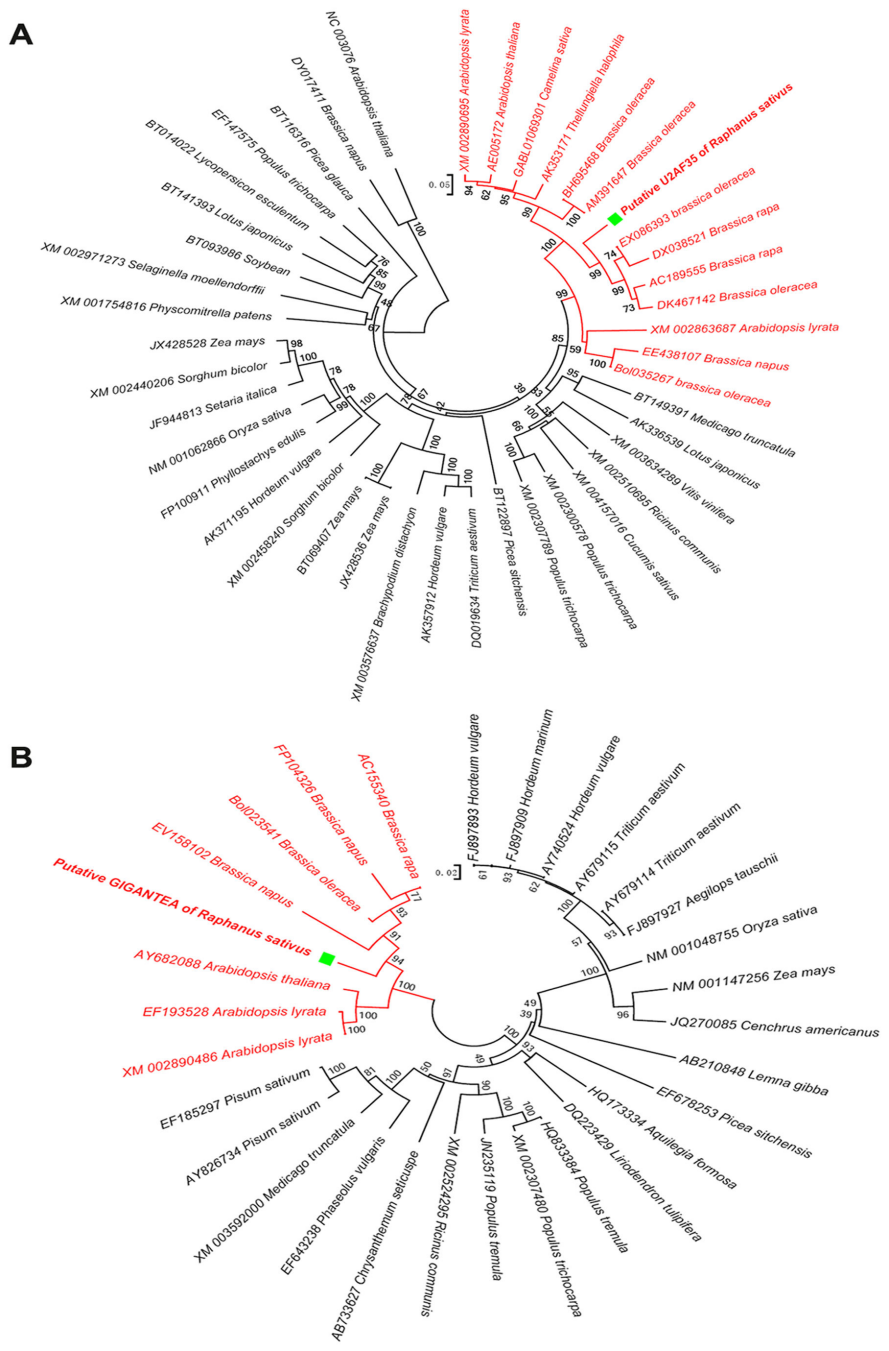
The phylogenetic tree analysis of *R. sativus* unigenes. The phylogenetic trees of U2AF35 and GIGANTEA unigenes in multiple plants were constructed using the neighbor-joining method. The *Brassicaceae* plants were denoted with red. Accordingly, (A) the U2AF35 unigene and (B) the GIGANTEA unigene of *R. sativus* were both marked with green square.

Some important genes that are widely involved in the processes of plant growth and development were further picked up for phylogeny analysis. KEGG analysis of the *R. sativus* unigenes revealed that many were involved in the spliceosome pathway (Table S4). U2AF35 is a component of the U2 snRNP Auxiliary Factor (U2AF), which is required for the recruitment of U2 snRNP to pre-mRNAs during spliceosome assembly [46]. Furthermore, U2AF35 is widely involved in many physiological processes in plants, including mitosis, photoperiodism, and flowering. Therefore, we aligned the U2AF35 unigene from the *R. sativus* transcriptome assembly with U2AF35 genes from multiple plants for which U2AF35 sequences were available in Genbank. Sequence alignment revealed that the U2AF35 gene was highly conserved among members of the *Brassicaceae*. A phylogenetic tree constructed on the basis of the sequence alignments demonstrated that the U2AF35 gene family can be divided into several groups in higher plants according to their nucleotide sequences. The sequence of the U2AF35 gene from *R. sativus* displays high homology to the U2AF35 sequences from *B. rapa* and *B. oleracea*. The U2AF35 gene can be roughly classified into four groups in higher plants, and the gene sequence identified in the transcriptome of *R. sativus* existed in the *Brassica* group. There are several species that can be found in the *Brassica* group such as *B. napus*, *Camelina sativa*, *A. thaliana* and *A. lyrata*. The U2AF35 gene from *R. sativus* shared 83% nucleotide identity with the same gene from *B. rapa* (accession no DX038521), 85.3% identity with the same gene from *B. oleracea* (accession no EX086393), 74.5% identity with the same gene from *A. thaliana* (accession no AE005172), and 68.9% identity with the same gene from *A.lyrata* (accession no XM002863687). Phylogenetic analysis based on multi-sequence alignment of the U2AF35 gene clearly shows that the acquired sequence from *R.sativus* resembles that from *B.rapa* and *B. oleracea* (Figure 5A).

Similar phylogenetic analysis was performed for the *GIGANTEA*, *ATIMD2*, and *EMB2369* unigenes from *R. sativus*. GIGANTEA promotes flowering under long days in a flowering pathway controlled by the circadian clock [47]. *ATIMD2* encodes the enzyme 3-isopropylmalate dehydrogenase, which is involved in leucine biosynthesis, whereas EMB2369 has ATP binding and aminoacyl-tRNA ligase activity. Multiple alignments of *GIGANTEA* genes revealed a high level of conservation of *GIGANTEA* sequences among *Brassicaceae* species. Similar to the phylogenetic analysis of *R. sativus* U2AF35, a *R. sativus GIGANTEA* sequence was grouped together with *GIGANTEA* sequences from *B. rapa*, *B. oleracea*, and *B. napus* (Figure 5B). The *GIGANTEA* gene can also be roughly classified into at least three groups in higher plants, with the gene identified from the present analysis of the *R. sativus* transcriptome existing in the *Brassica* group. The *GIGANTEA* gene from *R. sativus* shared 90.2% nucleotide homology with the same gene from *B. rapa* (accession no AC155340), 91.7% homology with the same gene from *B. oleracea* (accession no Bol023541), 88.3% homology with the same gene from *A. thaliana* (accession no AY2088), and 87.3% homology with the same gene from *A.lyrata* (accession no EF193528). Phylogenetic analyses of the *R. sativus ATIMD2* and *EMB2369* unigenes revealed similar results as those for the U2AF35 and *GIGANTEA* unigenes (Figure S3 and Figure S4). Taken together, phylogenetic analyses of the U2AF35, *GIGANTEA*, *ATIMD2*, and *EMB2369* unigenes uniformly demonstrated that *R. sativus* is highly homologous to members of the *Brassicaceae* species, including *B. rapa*, *B. oleracea* and *B.napus*. This might indicate that *R. sativus* and *B. rapa*, *B. oleracea* and *B.napus* share ancient kinship.

## Conclusions

Next-generation sequencing enabled *de novo* assembly of the transcriptome of *R. sativus*, an edible root vegetable that belongs to the *Brassicaceae family*. The assembly program Trinity generated 28,410 unigenes, which open the way for further research on gene expression patterns, functional genomics, and proteomics in *R. sativus*. Our characterization of the leaf transcriptome of *R. sativus* has not only enriched the publicly available database of sequences for members of the *Brassicaceae*, but will also facilitate genetic analysis of other non-model organisms. 

## Supporting Information

Figure S1 **Expression validation of *R.sativus* unigenes.** Five *R. sativus* unigenes were selected to perform RT-PCR assay. Result showed that these selected unigenes got right amplifications.
(TIF)

Figure S2 ***R. sativus* unigenes involved in alkaloid biosynthesis pathway.** *R.sativus* unigenes participating in the process of alkaloid biosynthesis derived from histidine and purine were marked with green rectangle.
(TIF)

Figure S3 **Phylogenetic tree analysis of ATIMD2 genes in multiple plants.** The phylogenetic tree of ATIMD2 unigenes in multiple plants were constructed using the neighbor-joining method. The *Brassicaceae* plants were denoted with red and ATIMD2 unigene of *R. sativus* was marked with green square.
(TIF)

Figure S4 **Phylogenetic tree analysis of EMB2369 genes in multiple plants.** The phylogenetic tree of EMB2369 unigenes in multiple plants were constructed using the neighbor-joining method. The *Brassicaceae* plants were denoted with red and EMB236 unigene of *R. sativus* was marked with green square.
(TIF)

Table S1 **The sequences of RT-PCR primers.** The sequences of primers used for validation of *R. sativus* unigenes were listed in Table S1.
(DOCX)

Table S2 **The information of four selected unigenes of *R. sativus*.** The accession numbers and the sequences of U2AF35, GIGANTEA, EMB2369 and ATIMD2 unigenes were included in Table S2.
(DOC)

Table S3 **Assembly summary of root unigenes of *R.sativus*.** The sequencing data of root of *R.sativus* was downloaded from NCBI Sequence Read Archive and assembled using Trinity. The assembly result was listed in Table S3.
(DOCX)

Table S4 **KEGG biochemical pathways of leaf unigenes of *R. sativus*.** In order to better understand the biological functions of *R. sativus* unigenes, a total of 7,158 unigenes were assigned to 117 KEGG biochemical pathways.
(XLSX)

Table S5 **KEGG biochemical pathways of top 10 GO terms of *R.sativus* leaf unigenes.** A total of 2,689 leaf unigenes (Top 10 GO terms) of *R.sativus* were assigned to 29 KEGG pathways.
(XLSX)

## References

[1] Watt J, Breyer-Brandwijk M (1962) The Medicinal and Poisonous Plants of Southern and Eastern Africa, 2nd edn, E. & S. Edinburgh and London, UK: Livingstone.

[2] Wang L, Wei L, Wang L, Xu C (2002) Effects of peroxidase on hyperlipidemia in mice. J Agric Food Chem 50: 868–870. doi:10.1021/jf011130 + PubMed : 11829659 11829659

[3] Xu L, Wang Y, Xu Y, Wang L, Zhai L et al. (2013) Identification and characterization of novel and conserved microRNAs in radish (*Raphanus* *sativus* L.) using high-throughput sequencing. Plant Sci 201-202: 108-114. doi:10.1016/j.plantsci.2012.11.010. PubMed: 23352408. 23352408

[4] Wang Y, Xu L, Chen Y, Shen H, Gong Y et al. (2013) Transcriptome Profiling of Radish (*Raphanus* *sativus* L.) Root and Identification of Genes Involved in Response to Lead (Pb) Stress with Next Generation Sequencing. PLOS ONE 8: e66539. doi:10.1371/journal.pone.0066539. PubMed: 23840502. 23840502 PMC3688795

[5] Schuster SC (2008) Next-generation sequencing transforms today’s biology. Nat Methods 5: 16–18. PubMed: 18165802. 18165802 10.1038/nmeth1156

[6] Shendure J, Ji H (2008) Next-generation DNA sequencing. Nat Biotechnol 26: 1135–1145. doi:10.1038/nbt1486. PubMed: 18846087. 18846087

[7] Wang Z, Gerstein M, Snyder M (2009) RNA-Seq: a revolutionary tool for transcriptomics. Nat Rev Genet 10: 57–63. doi:10.1038/nrg2484. PubMed: 19015660. 19015660 PMC2949280

[8] Wilhelm BT, Marguerat S, Watt S, Schubert F, Wood V et al. (2008) Dynamic repertoire of a eukaryotic transcriptome surveyed at single-nucleotide resolution. Nature 453: 1239–1243. doi:10.1038/nature07002. PubMed: 18488015. 18488015

[9] Cloonan N, Forrest Alistair RR, Kolle G, Gardiner BBA, Faulkner GJ et al. (2008) Stem cell transcriptome profiling via massive-scale mRNA sequencing. Nat Methods 5: 613–619. doi:10.1038/nmeth.1223. PubMed: 18516046. 18516046

[10] Feng C, Chen M, Xu CJ, Bai L, Yin XR et al. (2012) Transcriptomic analysis of Chinese bayberry (*Myrica* *rubra*) fruit development and ripening using RNA-Seq. BMC Genomics 13: 19. doi:10.1186/1471-2164-13-19. PubMed: 22244270. 22244270 PMC3398333

[11] Morin RD, Bainbridge M, Fejes A, Hirst M, Krzywinski M et al. (2008) Profiling the HeLa S3 transcriptome using randomly primed cDNA and massively parallel short-read sequencing. BioTechniques 45: 81-94. doi:10.2144/000112900. PubMed: 18611170. 18611170

[12] Shi CY, Yang H, Wei CL, Yu O, Zhang ZZ et al. (2011) Deep sequencing of the Camellia sinensis transcriptome revealed candidate genes for major metabolic pathways of tea-specific compounds. BMC Genomics 12: 131. doi:10.1186/1471-2164-12-131. PubMed: 21356090. 21356090 PMC3056800

[13] Tang Q, Ma XJ, Mo CM, Wilson IW, Song C et al. (2011) An efficient approach to finding *Siraitia* *grosvenorii* triterpene biosynthetic genes by RNAseq and digital gene expression analysis. BMC Genomics 12: 343. doi:10.1186/1471-2164-12-343. PubMed: 21729270. 21729270 PMC3161973

[14] Van Belleghem SM, Roelofs D, Van Houdt J, Hendrickx F (2012) De novo transcriptome assembly and SNP discovery in the wing polymorphic salt marsh beetle *Pogonuschalceus* (*Coleoptera,* *Carabidae*). PLOS ONE 7: e42605. doi:10.1371/journal.pone.0042605. PubMed: 22870338. 22870338 PMC3411629

[15] Vera JC, Wheat CW, Fescemyer HW, Frilander MJ, Crawford DL et al. (2008) Rapid transcriptome characterization for a nonmodel organism using 454 pyrosequencing. Mol Ecol 17: 1636–1647. doi:10.1111/j.1365-294X.2008.03666.x. PubMed: 18266620. 18266620

[16] Wang XW, Luan JB, Li JM, Bao YY, Zhang CX et al. (2010) De novo characterization of a whitefly transcriptome and analysis of its gene expression during development. BMC Genomics 11: 400. doi:10.1186/1471-2164-11-400. PubMed: 20573269. 20573269 PMC2898760

[17] Xiang LX, He D, Dong WR, Zhang YW, Shao JZ (2010) Deep sequencing-based transcriptome profiling analysis of bacteria-challenged Lateolabrax japonicus reveals insight into the immune-relevant genes in marine fish. BMC Genomics 11: 472. doi:10.1186/1471-2164-11-472. PubMed: 20707909. 20707909 PMC3091668

[18] Yang SS, Tu ZJ, Cheung F, Xu WW, Lamb JF et al. (2011) Using RNA-Seq for gene identification, polymorphism detection and transcript profiling in two alfalfa genotypes with divergent cell wall composition in stems. BMC Genomics 12: 199. doi:10.1186/1471-2164-12-199. PubMed: 21504589. 21504589 PMC3112146

[19] Grabherr MG, Haas BJ, Yassour M, Levin JZ, Thompson DA et al. (2011) Full length transcriptome assembly from RNA-Seq data without a reference genome. Nat Biotechnol 29: 644-652. doi:10.1038/nbt.1883. PubMed: 21572440. 21572440 PMC3571712

[20] Knowles DG, McLysaght A (2009) Recent de novo origin of human proteincoding genes. Genome Res 19: 1752-1759. doi:10.1101/gr.095026.109. PubMed: 19726446. 19726446 PMC2765279

[21] Li R, Zhu H, Ruan J, Qian W, Fang X et al. (2010) De novo assembly of human genomes with massively parallel short read sequencing. Genome Res 20: 265–272. doi:10.1101/gr.097261.109. PubMed: 20019144. 20019144 PMC2813482

[22] Ness RW, Siol M, Barrett SC (2011) De novo sequence assembly and characterization of the floral transcriptome in cross- and self fertilizing plants. BMC Genomics 12: 298. doi:10.1186/1471-2164-12-298. PubMed: 21649902. 21649902 PMC3128866

[23] Schulz MH, Zerbino DR, Vingron M, Birney E (2012) Oases: robust de novo RNA-seq assembly across the dynamic range of expression levels. Bioinformatics 28: 1086-1092. doi:10.1093/bioinformatics/bts094. PubMed: 22368243. 22368243 PMC3324515

[24] Simpson JT, Wong K, Jackman SD, Schein JE, Jones SJ et al. (2009) ABySS: a parallel assembler for short read sequence data. Genome Res 19: 1117–1123. doi:10.1101/gr.089532.108. PubMed: 19251739. 19251739 PMC2694472

[25] Zerbino DR, Birney E (2008) Velvet: algorithms for de novo short read assembly using de Bruijn graphs. Genome Res 18: 821-829. doi:10.1101/gr.074492.107. PubMed: 18349386. 18349386 PMC2336801

[26] Zhao QY, Wang Y, Kong YM, Luo D, Li X et al. (2011) Optimizing de novo transcriptome assembly from short-read RNA-Seq data: a comparative study. BMC Bioinformatics 12 (Suppl 14): S2. doi:10.1186/1471-2105-12-S1-S2. PMC3287467 22373417

[27] Barrero RA, Chapman B, Yang Y, Moolhuijzen P, Keeble-Gagnère G et al. (2011) De novo assembly of *Euphorbia* *fischeriana* root transcriptome identifies prostratin pathwayrelated genes. BMC Genomics 12: 600. doi:10.1186/1471-2164-12-600. PubMed: 22151917. 22151917 PMC3273484

[28] Miller HC, Biggs PJ, Voelckel C, Nelson NJ (2012) De novo sequence assembly and characterization of a partial transcriptome for an evolutionarily distinct reptile, the tuatara (*Sphenodon* *punctatus*). BMC Genomics 13: 43928. 10.1186/1471-2164-13-439 PMC3478169 22938396

[29] Sadamoto H, Takahashi H, Okada T, Kenmoku H, Toyota M et al. (2012) De novo sequencing and transcriptome analysis of the central nervous system of mollusk *Lymnaea* *stagnalis* by deep RNA sequencing. PLOS ONE 7: e42546. doi:10.1371/journal.pone.0042546. PubMed: 22870333. 22870333 PMC3411651

[30] Sun X, Zhou S, Meng F, Liu S (2012) De novo assembly and characterization of the garlic (*Allium* *sativum*) bud transcriptome by Illumina sequencing. Plant Cell Rep 31: 1823-1828. doi:10.1007/s00299-012-1295-z. PubMed: 22684307. 22684307

[31] Xu DL, Long H, Liang JJ, Zhang J, Chen X et al. (2012) De novo assembly and characterization of the root transcriptome of *Aegilops* *variabilis* during an interaction with the cereal cyst nematode. BMC Genomics 13: 133. doi:10.1186/1471-2164-13-133. PubMed: 22494814. 22494814 PMC3439707

[32] Zhang J, Liang S, Duan J, Wang J, Chen S et al. (2012) De novo assembly and characterisation of the transcriptome during seed development and generation of genic-SSR markers in peanut (Arachis hypogaeaL.). BMC Genomics 13: 90. doi:10.1186/1471-2164-13-90. PubMed: 22409576. 22409576 PMC3350410

[33] Conesa A, Gotz S, Garcia-Gornez JM, Terol J, Talon M et al. (2005) Blast2GO: a universal tool for annotation, visualization and analysis of functional genomics research. Bioinformatics 21: 3674–3676. doi:10.1093/bioinformatics/bti610. PubMed: 16081474. 16081474

[34] Kanehisa M, Goto S (2000) KEGG: Kyoto Encyclopedia of Genes and Genomes. Nucleic Acids Res 28: 27–30. doi:10.1093/nar/28.7.e27. PubMed: 10592173. 10592173 PMC102409

[35] Sharopova N, McMullen MD, Schultz L, Schroeder S, Sanchez-Villeda H et al. (2002) Development and mapping of SSR markers for maize. Plant Mol Biol 48: 463-481. doi:10.1023/A:1014868625533. PubMed: 12004892. 12004892

[36] Xu P, Wu X, Wang B, Liu Y, Ehlers JD et al. (2011) A SNP and SSR based genetic map of asparagus bean (*Vigna.* *unguiculata* *ssp.* *sesquipedialis*) and comparison with the broader species. PLOS ONE 6: 15952. doi:10.1371/journal.pone.0015952. PMC3017092 21253606

[37] Yu Y, Yuan D, Liang S, Li X, Wang X et al. (2011) Genome structure of cotton revealed by a genome-wide SSR genetic map constructed from a BC1 population between *gossypium* *hirsutum* and *G*. barbadense. BMC Genomics 12: 15. doi:10.1186/1471-2164-12-S3-S15. PubMed: 21214949. 21214949 PMC3031231

[38] Larkin MA, Blackshields G, Brown NP, Chenna R, McGettigan PA, McWilliam H, Valentin F, Wallace IM, Wilm A, Lopez R, Thompson JD, Gibson TJ, Higgins DG (2007) Clustal W and Clustal X version 2.0. Bioinformatics 23: 2947-2948. doi:10.1093/bioinformatics/btm404. PubMed: 17846036. 17846036

[39] Tamura K, Dudley J, Nei M, Kumar S (2007) MEGA4: molecular evolutionary genetics analysis (MEGA) software version 4.0. Mol Biol Evol 24: 1596-1599. doi:10.1093/molbev/msm092. PubMed: 17488738. 17488738

[40] Góngora-Castillo E, Buell CR (2013) Bioinformatics challenges in de novo transcriptome assembly using short read sequences in the absence of a reference genome sequence. Nat Prod Rep 30: 490-500. doi:10.1039/c3np20099j. PubMed: 23377493. 23377493

[41] Wang X, Wang H, Wang J, Sun R, Wu J et al. (2011) The genome of the mesopolyploid crop species *Brassica* *rapa* . Nat Genet 43: 1035-1039. doi:10.1038/ng.919. PubMed: 21873998. 21873998

[42] Kamei A, Tsuro M, Kubo N, Hayashi T, Wang N et al. (2010) QTL mapping of clubroot resistance in radish (*Raphanus* *sativus* *L*.). Theor Appl Genet 120: 1021–1027. doi:10.1007/s00122-009-1230-z. PubMed: 20012934. 20012934

[43] Nakatsuji R, Hashida T, Matsumoto N, Tsuro M, Kubo N et al. (2011) Development of genomic and EST-SSR markers in radish (*Raphanus* *sativus* . p. L). Breed Sci 61:413-9 10.1270/jsbbs.61.413 PMC3406777 23136479

[44] Ohsako T, Hirai M, Yamabuki M (2010) Spatial structure of microsatellite variability within and among populations of wild radish *Raphanus* *sativus* L. var. *hortensis* Backer f. *raphanistroides* Makino (*Brassicaceae*) in Japan. Breed Sci 60: 195–202. doi:10.1270/jsbbs.60.195.

[45] Yamane K, Lü N, Ohnishi O (2009) Multiple origins and high genetic diversity of cultivated radish inferred from polymorphism in chloroplast simple sequence repeats. Breed Sci 59: 55–65. doi:10.1270/jsbbs.59.55.

[46] Zamore PD, Green MR (1989) Identification, purification, and biochemical characterization of U2 small nuclear ribonucleoprotein auxiliary factor. Proc Natl Acad Sci U_S_A 86: 9243–9247. doi:10.1073/pnas.86.23.9243. PubMed: 2531895. 2531895 PMC298470

[47] Sawa M, Kay SA (2011) GIGANTEA directly activates Flowering Locus T in *Arabidopsis* *thaliana* . Proc Natl Acad Sci U_S_A 108: 11698-11703. doi:10.1073/pnas.1106771108. PubMed: 21709243. 21709243 PMC3136272

