# Supplementary figures and images for: Transcriptome Analysis of Leaf Tissue of Raphanus sativus by RNA Sequencing
Source: PLoS One. 2013 Nov 12;8(11):e80350. doi: 10.1371/journal.pone.0080350 (PMC3827192; doi:10.1371/journal.pone.0080350)

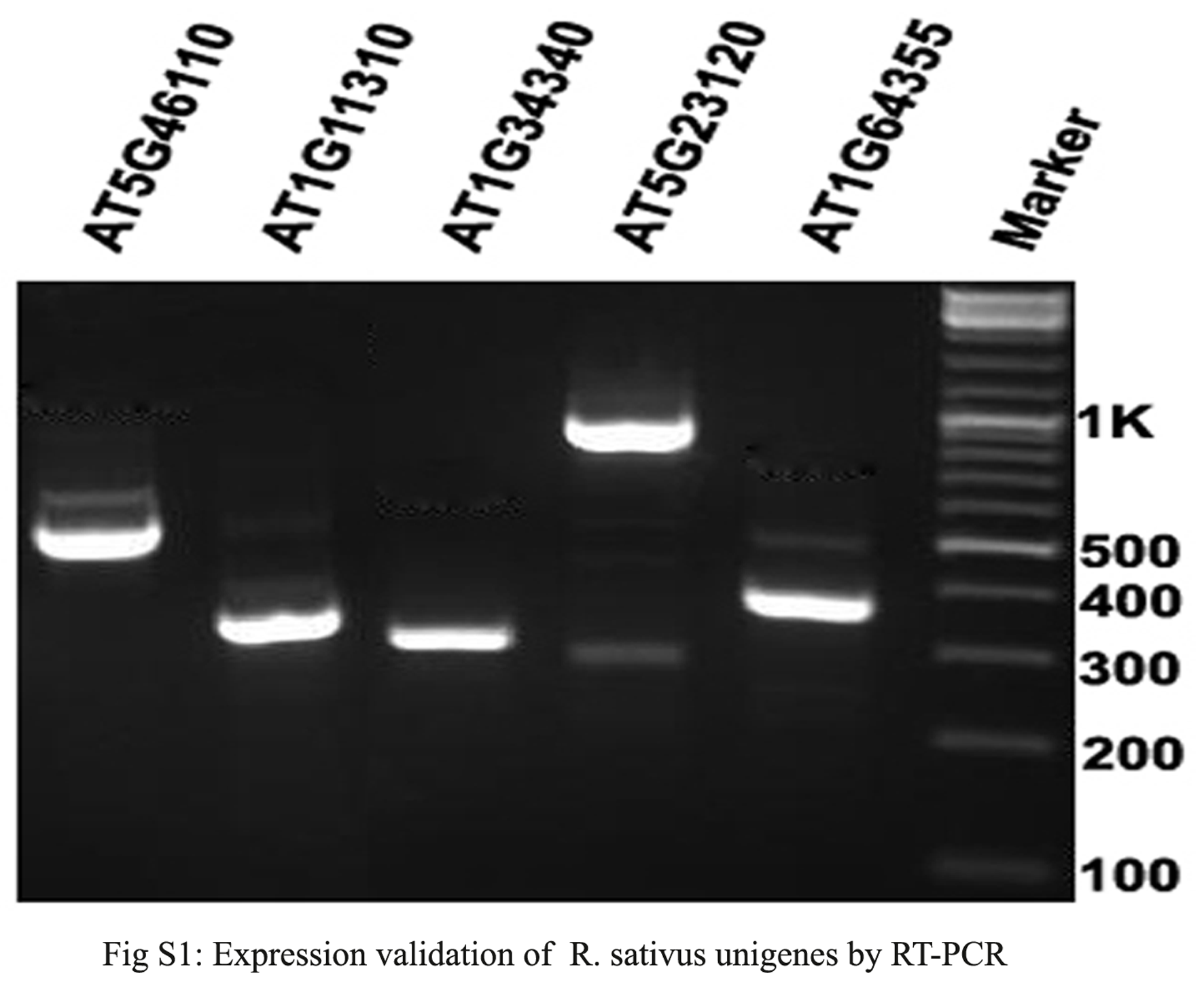

Supplement: Figure S1 — Expression validation of R.sativus unigenes. Five R. sativus unigenes were selected to perform RT-PCR assay. Result showed that these selected unigenes got right amplifications. (TIF) [file pone.0080350.s001.tif]

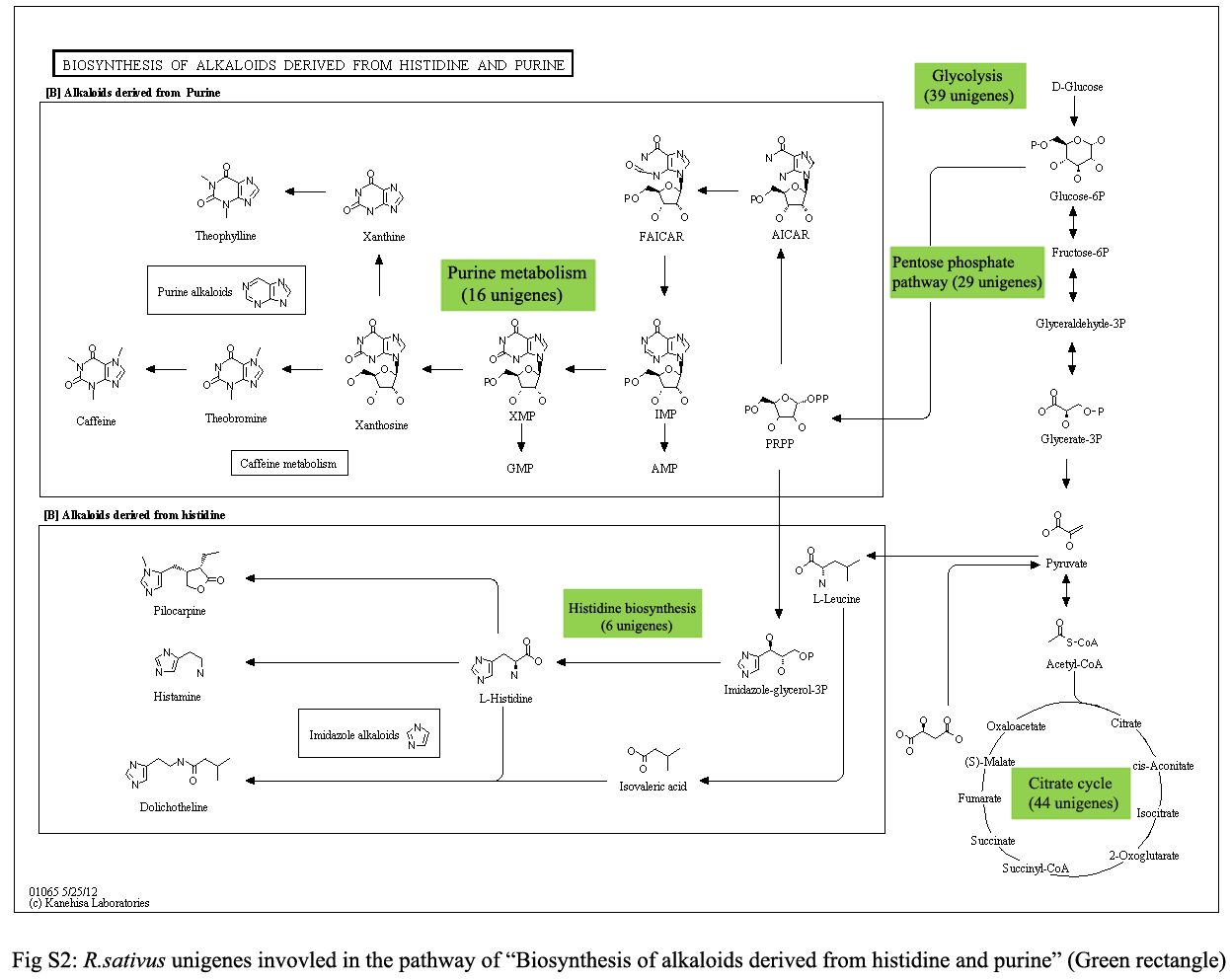

Supplement: Figure S2 — R. sativus unigenes involved in alkaloid biosynthesis pathway. R.sativus unigenes participating in the process of alkaloid biosynthesis derived from histidine and purine were marked with green rectangle. (TIF) [file pone.0080350.s002.tif]

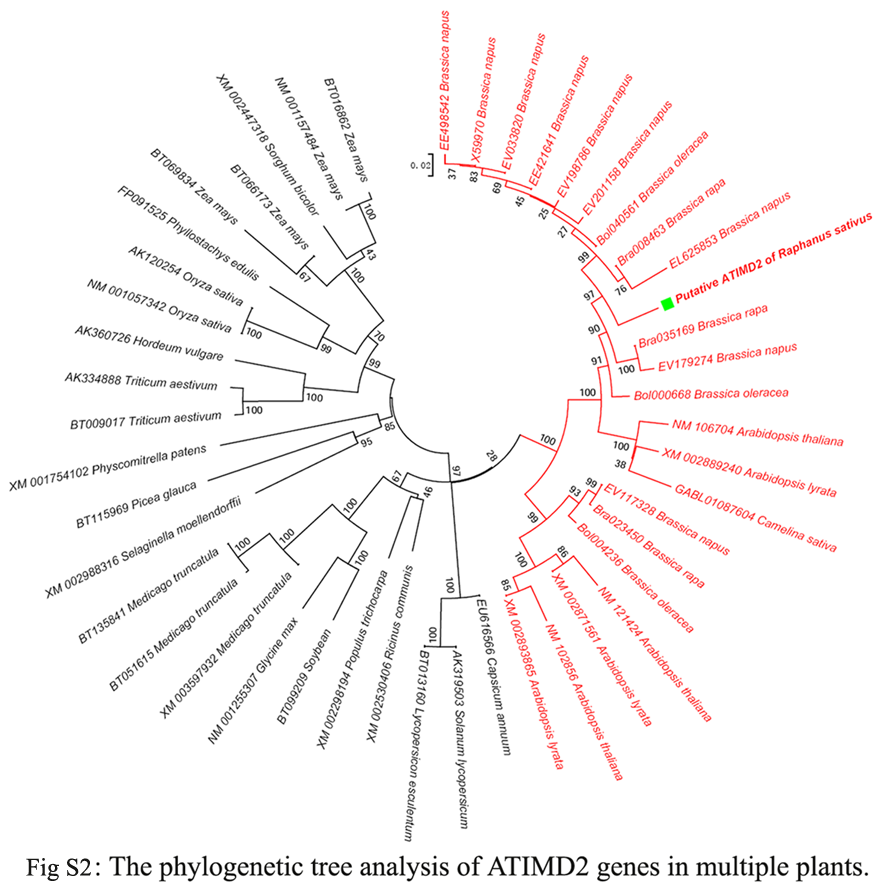

Supplement: Figure S3 — Phylogenetic tree analysis of ATIMD2 genes in multiple plants. The phylogenetic tree of ATIMD2 unigenes in multiple plants were constructed using the neighbor-joining method. The Brassicaceae plants were denoted with red and ATIMD2 unigene of R. sativus was marked with green square. (TIF) [file pone.0080350.s003.tif]

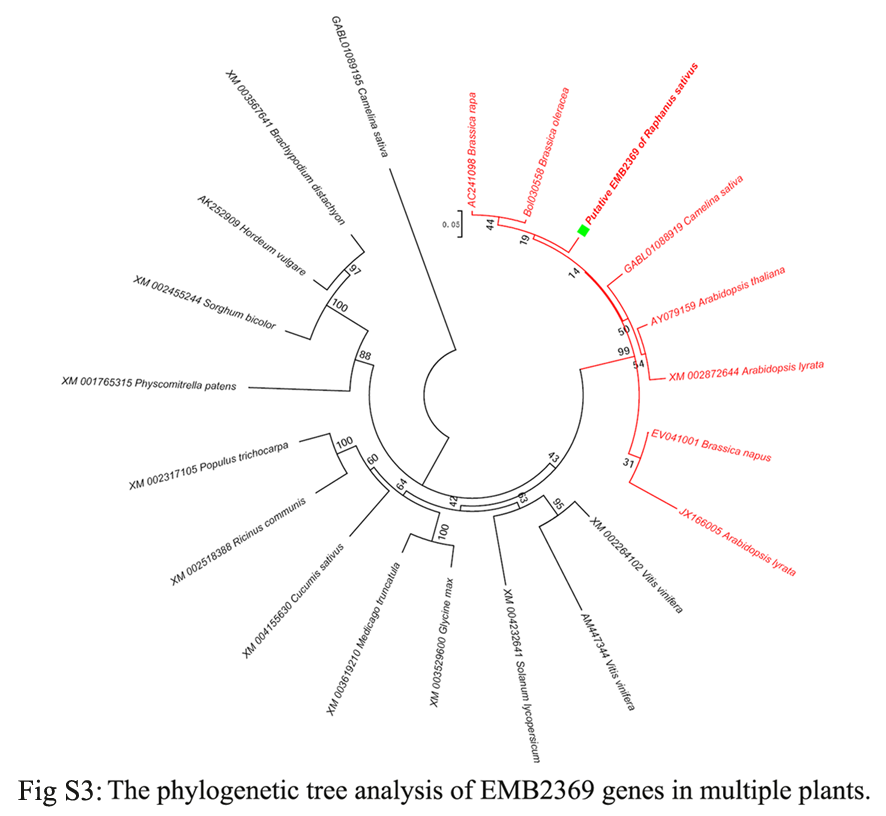

Supplement: Figure S4 — Phylogenetic tree analysis of EMB2369 genes in multiple plants. The phylogenetic tree of EMB2369 unigenes in multiple plants were constructed using the neighbor-joining method. The Brassicaceae plants were denoted with red and EMB236 unigene of R. sativus was marked with green square. (TIF) [file pone.0080350.s004.tif]
