# Supplementary material for: Transcriptome Analysis of Leaf Tissue of Raphanus sativus by RNA Sequencing
Source: PLoS One. 2013 Nov 12;8(11):e80350. doi: 10.1371/journal.pone.0080350 (PMC3827192; doi:10.1371/journal.pone.0080350)
Supplement: Table S1 — The sequences of RT-PCR primers. The sequences of primers used for validation of R. sativus unigenes were listed in Table S1. (DOCX) [file pone.0080350.s005.docx]

| **Table S1. PCR primers used for validations of assembled *Raphanus sativus* Unigenes** | | | |
| --- | --- | --- | --- |
| **Accession** **(*Raphanus sativus*)** | **ID(*Arabidopsis thaliana*)** | **Forward Primer** | **Forward Primer** |
| comp28780_c0_seq1 | AT5G46110 | CGTCACCGGATTCTTCTTCTT | CCGAGTACAACAGGAGCTAAAG |
| comp28781_c0_seq1 | AT1G11310 | CTCTCGGAAAGACCAAGATGAG | GCACGGCTACAAACCAGATA |
| comp26435_c0_seq1 | AT1G34340 | CTGGAGGAGCCTTCGATAAAC | TCAGGATTTGGAGGCTGTTATG |
| comp14104_c0_seq1 | AT5G23120 | CTCCTCCACCATCTTCATCTTC | CTTCTTCCTCTGAGCGATAACC |
| comp19347_c0_seq1 | AT1G64355 | CTGAGACCCAACACGATGAA | ACACTTGACAGAGACGAAAGAG |
