# Supplementary material for: Transcriptome Analysis of Leaf Tissue of Raphanus sativus by RNA Sequencing
Source: PLoS One. 2013 Nov 12;8(11):e80350. doi: 10.1371/journal.pone.0080350 (PMC3827192; doi:10.1371/journal.pone.0080350)
Supplement: Table S2 — The information of four selected unigenes of R. sativus. The accession numbers and the sequences of U2AF35, GIGANTEA, EMB2369 and ATIMD2 unigenes were included in Table S2. (DOC) [file pone.0080350.s006.doc]

**comp20129_c0_seq1:**

TTTGGGAATGTGTATGTTCAGTTTAAGGAGGAGGATCAGGCGGCCGCGGCTTTGAAGGCC

TTGGAAGGGAGGAGTTACTTGGGCCGGCCCATTATCGCGGAGTTTTCGCCTGTGACGGAT

TTTAGGGAGGCGACGTGTAGGCAGTATGAGGAGGATAACTGTGGGCGCGGTGGGTATTGT

AATTTTATGCATGTGAAGCTTGTTTCGAGGGAGATGAGGAGGAAGCTGTTTGGGAGGAGG

AGGTCGTACCGTAGGGGAAGCAGGAGTAGGAGTAGGAGTGTGAGCCCCAGGGGGAAGAGA

GAGTATGACCGGCGTGGTGATCGTGAACCTCCTCGCAGGGGGGAGTTTGGTCACCGGGAC

AGAGATGGGGAGTTTTATAGGCATGGAAGTGGGAGAAGAAGGAGTGAGAGAGGAGACAGG

GACGGTTATTCTAGGAGGAGACGTGAGGGAAGCCCTGGTGGTGGTGGGAGAGAAGGAAGC

GAGGAGAGGAGGGCAAGGATTGAGCAATGGAACAGGGAACGGGAGGAGAAGGAAGAGGGA

GAAG

**comp7714_c0_seq1:** CGGAAAAGTTCTAAAAAGAGAGAATGGACCATAGCACGAAGCTCAGCCATAGCGATGTTC

TTGGCCGCTGATCTCGTGGTTAAACTCGATCTAGCTTTCCTGATACAAGTCTCCGGCGGA

AAAGTTCTAGTGAGGATTGTTTCCACGGTGGCAACAAATATTTTCATCAAGCATGCCTCG

GAAGGGCTCCCACGTGGAAGATATTCAAGTACTTTGAGGAGTGGGATGTATAGATTCCAT

GACAAGATGGGAGGTTGCAGTGGGGTGGCAACAATGATCTCTGGAAGATCAACCGCTGAG

GAGTTTAAAGGGATCAATCCATAGGCAGCTTCCCATATAGTGCAGATTCTCCACTCCACT

TCAGGACCATGAGCACAAAGCATCGAGGCGATTCCTTGAGCAGTGGCTTCAATGGTTGCT

TCTG

**comp11084_c0_seq1:** TCGAAAAATCCAGCTAATGGGGATGCTATTCCCATATGGGTAGCTGATTATGTTCTAGCA

AGCTATGGAACCGGAGCAATCATGGCCGTCCCAGCTCATGATACTCGGGATAATGAGTTT

GCGTTAAAGTATAATATTCCAATCAAGTGGGTAGTGAGGAACGAGGCAAGTTCAAGTGAT

GATGCTAAGCAGGTTTATGCAGGAGTGGGAGTTATTGAAAACTCATCAAGTGTGGAAACA

GGACTTGACATTAATCAACTATCTAGCAAAGAAGCTGCTTTGAAAGTTATTGAATGGGCT

GAGAGAACAGGAAATGGAAAGAAAAAGGTAAATTACAAGTTGAGAGACTGGCTATTTGC

ACGGCAGCGTTACTGGGGTGAACCTATCCCTATCTTAATCTTGGACGAGTCTGGTGAAACT

ATTG

**comp28243_c0_seq1:**

AACACGCCAAAAGTTTCAATGTGATCAAAATACGTCCCGCTAATATAACATACATTGTGG

CTGATGAGACTCTGATATTCTCTTCGCGTACAAACACTTATTATTATTGGAAAAATAATG

AAAATTCTCCTCAAAGTTGTACAGATGATTAAACAGAAGCTTGAACATGGGAGTCCACTG

ACTTCAGAACCTCCTCTCCCATCTCCTTGCACCCCACAAGTTTAGTTCCTGCGGAGTAGA

TGTCTCCTGTTCTGAATCCTTTGTTCAGAGCACCCAACACCGCGTCTTCAATTCTCTTAG

CTGCCTTTTCTTCTCCAAGTCCGTATTTCAGAAGCATCGCAGCGCTGAGGATGGTTGCCA

ACGGGTTCGCTTTATCCTGTCCAGCAATATCAGGTGCGGAGCCATGTATAGGTTCAAAGA

GTCCAGGTCCCGAATCACTGAGACTAGCAGAGGGAAGCATGCCAATGCTTCCTGTGATCA

TTGACGCTTCATCAGATAATATATCCCCAAAAATGTTGTTTGTGACAATGGTGTCAAACT

GTTTAGGGTCACGAACAAGCTGCATTGCAGCATTGTCAACATACATATGTGACAGCTCAA
