# Supplementary material for: Transcriptome Analysis of Leaf Tissue of Raphanus sativus by RNA Sequencing
Source: PLoS One. 2013 Nov 12;8(11):e80350. doi: 10.1371/journal.pone.0080350 (PMC3827192; doi:10.1371/journal.pone.0080350)
Supplement: Table S3 — Assembly summary of root unigenes of R.sativus. The sequencing data of root of R.sativus was downloaded from NCBI Sequence Read Archive and assembled using Trinity. The assembly result was listed in Table S3. (DOCX) [file pone.0080350.s007.docx]

| **Table S3. Summary of assembly of root unigenes of *Raphanus sativus*** | |
| --- | --- |
| **Assembly** | **Number** |
| Number of used reads | 26,381,880 |
| Total Unigenes generated | 50,700 |
| N_50_ length (bp) | 1623 |
| Average Unigene length (bp) | 1181 |
| shortest unigene Length (bp) | 200 |
| longest unigene Length (bp) | 15819 |
